# Implementation of a parent training intervention (SPARCK) to prevent childhood mental health problems: study protocol for a pragmatic implementation trial in Norwegian municipalities

**DOI:** 10.1186/s13063-024-08704-7

**Published:** 2024-12-21

**Authors:** Anette Arnesen Grønlie, Agathe Backer-Grøndahl, Ragnhild Bang Nes, Maria Begoña Gomez, Truls Tømmerås

**Affiliations:** 1 Norwegian Center for Child Behavioral Development, Oslo, Norway; 2 Promenta Research Center, Department of Psychology, University of Oslo, Oslo, Norway; 3 Norwegian Institute of Public Health, Oslo, Norway

**Keywords:** Implementation, Implementation determinants, Implementation outcomes, Co-creation, Intervention, Child mental health problems, Implementation strategies, Protocol

## Abstract

**Background:**

Effective evidence-based interventions (EBI) are necessary to prevent and avoid negative life trajectories for children with mental health problems. Even though many EBIs prove effective when tested, few are successfully implemented and used in real-world clinical practice. As a result, many children and families do not receive the best care in due time or at all. To reduce this research-practice gap, a combined RCT and implementation study of Supportive Parents—Coping Kids (SPARCK), a parent training intervention to prevent childhood mental health problems, will be performed. This study protocol concerns the implementation part of the larger effectiveness-implementation project.

**Methods:**

The study is a correlational multi-site implementation study of SPARCK performed alongside a two-armed RCT, in 24 Norwegian municipalities. A quantitative three-wave longitudinal web-based data collection will be conducted among SPARCK practitioners and leaders in relevant services. We will investigate the relations between theory-driven and empirical implementation determinants and implementation outcomes, measured by fidelity, acceptability, appropriateness, and feasibility. In addition, we will examine how these implementation determinants and outcomes are associated with the clinical outcomes of SPARCK.

**Discussion:**

The current study will investigate implementation determinants and their relation to indicators of implementation success, while simultaneously investigating effectiveness of an intervention optimized to the needs of both the target group and relevant stakeholders. Together, this may improve clinical effect, contextual fit, implementation success, and reduce the time lag between research findings and application in real-world settings.

**Trial registration:**

ClinicalTrials.gov NCT05800522. Registered on 2023.03.23.

**Supplementary Information:**

The online version contains supplementary material available at 10.1186/s13063-024-08704-7.

## Contributions to the literature

- By conducting an implementation study alongside an effectiveness trial, this project may contribute with knowledge on how to accelerate implementation of EBIs into practice and disentangle the relations between implementation outcomes and clinical outcomes.
- This study will add to the implementation science by generating much-needed knowledge about the impact of implementation determinants on specific implementation outcomes (fidelity, acceptability, appropriateness, and feasibility).
- The current study capitalizes on a series of implementation strategies used in a previous development and optimization phase. As such, the study will contribute to the field of implementation strategies by documenting activities needed to promote implementation outcomes.

## Background

During the latest half-century, numerous interventions aimed at reducing internalizing and externalizing mental health problems in children have been developed and tested empirically [1]. Undoubtedly, early identification and effective evidence-based interventions (EBIs) to prevent and reduce these problems are important [1–4]. Although several EBIs have proven effective in clinical research, they are rarely implemented and used in routine practice. A major challenge is that attempts to implement EBIs into routine practice often fail [5] or take too long. In fact, the time lag between production of evidence to practical application still averages to 15 years and more [6–9]. If an intervention is not implemented successfully, it will not be effective in the practice field regardless of its clinical effectiveness [5]. As a result, individuals in need of intervention will not benefit from them [10]. The lack of effective EBIs in routine practice is a challenge in Norway as well, where most children and families in need do not have access to them [11–13]. This failure to implement clinically effective interventions in routine practice is referred to as the research-practice gap between scientific knowledge and its application in real-world settings. The ultimate goal of implementation science is to reduce this gap and ensure that the best interventions for a particular target group become available and standard care in all relevant settings [14].

As such, to enhance the uptake, reach, and sustained use of EBIs aimed at preventing and reducing internalizing and externalizing mental health problems in children, greater attention should be devoted to research on the implementation of such EBIs. Traditionally, there has been a substantial focus in the implementation field on studying individual, process, and system level determinants in the sites where the EBIs are introduced and implemented [15]. Studying such contextual implementation determinants is an integral part of identifying and accounting for implementation outcomes [16, 17]. However, it has recently been suggested that to fully understand and improve implementation and sustainment of EBIs, more focus should be directed towards studying intervention characteristics and their relation to implementation outcomes. Even more crucial is to ensure that the EBIs cater to the unique needs of both users and stakeholders [15, 18]. When developing a new intervention, it is essential to focus on the priorities, preferences, and status quo of individuals and service settings where the intervention will be implemented. User-centered design (UCD) is a product development approach rooted in human–computer interaction, which has recently emerged in the field of psychosocial intervention design. The approach emphasizes end-user needs, prototyping and rapid iterations, redesign and simplification, as well as exploitation of natural constraints [15]. The goal of a UCD process is to enhance an intervention’s usability, acceptance, user satisfaction, and ultimately to improve its external validity and effectiveness. As such, an important focus of the overall Supportive Parents—Coping Kids (SPARCK) project has been to apply a UCD approach from the development phase of the SPARCK intervention. More specifically, the current study builds upon an elaborate co-creation and optimization period of not only the intervention, but also key implementation strategies.

### Co-creation of SPARCK intervention

What follows provides a brief overview of the optimization phase and study of the SPARCK intervention (see protocol for intervention development [19]). SPARCK is a novel transdiagnostic parent training intervention aimed at preventing mental health problems in children [19–21]. It is tailored to frontline level services in Norwegian municipalities. The target group consists of parents of children aged 4 to 12 with elevated, but sub-clinical levels of internalizing symptoms (i.e., anxiety, depression, and withdrawal) and/or externalizing symptoms (i.e., conduct problems, opposition, and cooperation challenges). The intervention consists of a maximum of 12 sessions delivered individually and contains well-established and effective elements which are tailored to help parents promote coping skills in their children (see protocol for RCT study [21]).

The process of creating and refining SPARCK drew inspiration from three complementary design approaches: the IDEAS Impact Framework, which guides in creation and evaluation of program components [22, 23]; the Multiphase Optimization Strategy (MOST), a framework for optimization of intervention content [24]; and UCD, which ensures that the intervention remains responsive to the needs and preferences of its intended users throughout the development process [15]. A prototype of SPARCK was developed and piloted in three families (2018–2019). From the start, the implementation strategy “co-creation” was used actively, to increase usability, contextual fit, and implementability [18, 19, 25, 26]. In Norway, potential target families for SPARCK encounter the municipal services across various systems, such as school health, educational and psychological counseling services, and child welfare services. Thus, in addition to tailoring the intervention to each family and their challenges, it was also important to adapt and optimize the implementation to the different services delivering it.

After the pilot test, seven municipalities were recruited, and 14 practitioners, two from each municipality, were trained in a modified version of SPARCK. The modifications were conducted based on feedback from the pilot families and the practitioners’ experience. This was the first wave in the optimization process, which continued with a study consisting of two iterative mixed-methods cycles (2020–2021), in which practitioners tested SPARCK with 14 families in both cycles. After each cycle, SPARCK was optimized based on feedback from families and practitioners [19]. The clinical efficacy of the intervention in terms of reduction in child internalizing and externalizing symptoms, as well as increase in positive parent–child interaction, was tested using single case experimental designs (SCEDs) with quantitative assessments at multiple time points. In addition, qualitative semi-structured interviews with parents were conducted post intervention [19, 20].

Alongside the iterative SCEDs and parent interviews, we conducted an exploratory implementation study with semi-structured interviews with the practitioners and their leaders in the seven municipalities in order to conduct a contextual analysis of determinants to successful implementation of SPARCK [27]. The aim was to gain insights from the relevant stakeholders on barriers and facilitators to implementation, to inform intervention optimization and aid choice and adaptation of implementation strategies to overcome identified barriers [28]. Furthermore, contextual analyses can be used to understand and interpret subsequent effectiveness and implementation outcomes [29], and as such, the secondary aim was to use the contextual analysis as a segue to the current RCT [21] and implementation study. The Consolidated Framework for Implementation Research (CFIR) [30, 31] is a comprehensive framework, designed to guide and enhance implementation research. It provides a structured approach for identifying and assessing potential barriers and facilitators to successful implementation of novel innovations. In the qualitative implementation study, CFIR was used to construct the interview guides, identify, analyze, and report on implementation determinants. Based on these findings and a recently developed implementation measure [32–34], a quantitative measure on determinants to implementing change was customized to SPARCK and the study context, with the aim of using it in the current implementation study.

### Implementation outcomes, determinants, and strategies

To differentiate between intervention and implementation success or failure, it is imperative to assess implementation outcomes independently, alongside clinical outcomes [5, 35], while also disentangling their distinct contributions. In the present study, four implementation outcomes will be studied, and according to Damschroder et al. [36], these can be regarded as anticipated implementation outcomes and predictors of actual implementation outcomes. One such outcome that will be studied is fidelity. Historically, fidelity has been measured and reported more often than other implementation outcomes, and typically describes the degree of adherence to an intervention protocol, dose, and quality of delivery [5]. Other outcomes in the current study, which are important in the early stages of all implementation efforts, include acceptability, appropriateness, and feasibility. While acceptability is the perception that an intervention is agreeable, palatable, or satisfactory, appropriateness can be described as the perceived fit, relevance, or compatibility of an intervention for a given setting or to address a particular problem [5]. Lastly, feasibility is the extent to which stakeholders believe a new intervention can be successfully used or carried out within a given setting [5].

Whereas implementation outcomes evaluate the degree of implementation success, and serve as prerequisites of clinical outcomes, implementation determinants may be defined as barriers and facilitators that influence the implementation outcomes [17]. To advance the field of implementation, a need for more clarity on the relations between determinants and specific implementation outcomes has been expressed [10]. Several implementation determinants have proven to be important precursors for successful implementation [17, 30, 31], including organizational factors such as readiness [37, 38]. Organizational readiness refers to organizational members’ collective change commitment and change efficacy to implement a certain change and can be more or less present in an individual, group, unit, department, or organization [38]. According to Damschroder et al. [36], CFIR now views implementation readiness as a higher-order construct within the framework.

Deciding on how to address and overcome identified contextual barriers is another fundamental challenge in implementation research. Implementation strategies are the “how to” part of implementation and can be defined as specific methods or techniques used to improve the adoption, implementation, and sustainment of an intervention in the practice field [39, 40]. The current study capitalizes on a series of implementation strategies utilized in the co-creation and optimization phase of the intervention. These strategies are based on data from the iterative cycles, semi-structured interviews and feedback from relevant stakeholders [19, 27], as well as existing knowledge in the field [25, 26]. Ultimately, the current study will use a variety of discrete and multifaceted implementation strategies [25, 26, 39] (see Additional file 1), chosen not only for the purpose of the current study but also for future implementation and sustainment in the practice field [18]. Implementation strategies entail extensive resources; however, these are often not reported prospectively and are typically investigated as part of implementation studies after an intervention has demonstrated effectiveness. In addition to delaying the implementation into real-world settings, this is a missed opportunity for furthering the implementation science field [41] and a possible source for replicability problems for both research and practice [42].

## Methods

### Aim

This study is part of a larger effectiveness-implementation trial of the parent training intervention SPARCK. In a randomized controlled trial (RCT), SPARCK’s clinical effectiveness in preventing and reducing negative outcomes and promoting positive outcomes in children and parents is investigated. A separate study protocol is written for the RCT [21]. In order to grant appropriate attention and space to the implementation part of the project, a separate implementation study protocol was deemed warranted. In the current study, we aim to investigate the relations between implementation determinants [30, 31] and successful implementation of SPARCK, measured by four key implementation outcomes, namely fidelity, acceptability, appropriateness, and feasibility [5]. In addition, we examine how implementation determinants and implementation outcomes are associated with clinical outcomes of the intervention. More specifically, we have four research hypotheses to guide investigation of the implementation of SPARCK: (H1) positive ratings of implementation determinants are associated with better implementation outcomes; (H2) positive ratings of implementation determinants are associated with better clinical outcomes for parent and child; (H3) better implementation outcomes are associated with better clinical outcomes for parent and child; (H4) implementation determinants and outcomes are rated more positively with increasing experience with SPARCK (i.e., over time). For this last hypothesis, we will also investigate the bi-directional relations between implementation determinants and implementation outcomes over time.

In addition to these hypotheses, we will explore whether practitioners and leaders from the municipalities that engaged in the co-creation process of SPARCK report higher initial levels of organizational readiness than those from municipalities recruited directly to the combined RCT and implementation study. Finally, the implementation strategies ultimately used, modified, added, or omitted in the trial will be specified and reported in a separate article [25, 39]. Collectively, these efforts may lead to improved clinical outcomes when SPARCK is implemented in clinical settings, along with better contextual fit, greater implementation success, and a reduced time lag between research findings and their application in practice [18, 43, 44].

### Study design and data collection

This study is a correlational implementation study performed alongside a two-armed RCT [21]. The combined study can be described as a hybrid effectiveness-implementation type 2 trial [43, 44], although the implementation strategies are not tested per se. To test the four research hypotheses, we will include a quantitative longitudinal web-based data collection spanning three time points, among SPARCK practitioners and their leaders. The first time point (T1) was during the initiation of the intervention in 2023, shortly after training of practitioners and information dissemination to leaders. The second data collection (T2) was after approximately 1 year when the practitioners had completed two cases each. The last data collection (T3) will be conducted after approximately 2 years (2025), when the practitioners have completed four cases each. In addition, for hypotheses H2 and H3 concerning relations with clinical outcomes, we will also include parent reported data on child symptoms and parent–child interaction from the RCT at pre, post (treatment termination), and follow-up (6 months after post) [21].

### Setting, participants, and recruitment

The study will be conducted in 24 municipalities, which vary in size, demography, and urbanicity and together represent all five health regions in Norway. Efforts are made to recruit heterogeneous municipalities, to reflect conditions as they are in the real world. The municipalities are part of an implementation network hosted by the Norwegian Center for Child Behavioral Development (NUBU; Norwegian acronym), and thus have some experience with implementation of EBIs [45]. Of the 24 municipalities, seven participated in co-creation of SPARCK [19].

Participants in the implementation study are SPARCK practitioners and municipal leaders. Currently, this includes 44 recruited practitioners, including 14 from the seven co-creation municipalities. In addition to training in SPARCK, all practitioners are trained in and actively practice Parent Management Training—Oregon model (PMTO) [45, 46]. Some are also trained in other EBIs. All practitioners are employed in municipal frontline mental health services, such as health care, school health services, and child welfare services. Participants also include the practitioners’ leaders, including their immediate manager, and superior leaders at mid and top managerial levels in the various services. Around 80 municipal leaders have so far been identified and recruited to the study. However, as staff turnovers are bound to happen, more will be recruited as needed. Written informed consent will be obtained from all participants before inclusion in the study.

### Implementation frameworks and theory

Using a robust theoretical framework is essential for elucidating the mechanisms that underpin the success or failure of intervention implementation, as well as for identifying the factors that influence intervention outcomes [17]. The study will be guided by the determinant framework CFIR [30, 31], which will be used to systematically specify and assess potential barriers and facilitators along its five domains (innovation, outer setting, inner setting, individuals, and implementation process). These domains, along with their underlying constructs, will help explain how various determinants influence the implementation outcomes. The implementation theory Organizational Readiness [38] provides a theoretical foundation for understanding the relationship between readiness and implementation outcomes. This theory conceptualizes organizational readiness as a shared team attribute, making it particularly suited for organizational changes that require collective and coordinated behavior change for effective implementation and realization of anticipated benefits [38]. Proctor et al. have developed a comprehensive Implementation Outcomes Framework that delineates eight distinct implementation outcomes [5]. The current study will focus on evaluating four key implementation outcomes from this framework that are of importance to SPARCK. Lastly, the Implementation Research Logic Model (IRLM) [47] is a conceptual framework that maps out the interconnections among various components of an implementation project, providing both a rationale and a roadmap for the project’s execution. In the present study, the IRLM will be used as an analytical tool to clarify and specify the hypothesized relations among key elements, including implementation determinants, strategies, mechanisms of action, implementation outcomes, and intervention outcomes.

### Measures

All measures in the current study are Norwegian versions, which have been adapted to the current context with permissions from the original authors and have undergone piloting with similar individuals to the study sample. Measures on clinical outcomes are described in detail in the RCT protocol [21] and include various standardized and widely used instruments to investigate parent reported change in child externalizing and internalizing symptoms.

#### Background variables

Demographic and service-related variables will be obtained from all participants (i.e., age, gender, municipality, type of employment service, educational level, professional background, role, job experience, training in EBIs, counseling experience, familiarity with SPARCK).

#### Implementation outcomes

Hypotheses H1, H3, and H4 will be tested using various measures of implementation outcomes. The Fidelity Questionnaire (FidQ) will be employed to assess practitioners’ fidelity to SPARCK. FidQ was initially developed during the intervention’s design phase and refined prior to this study based on practitioner feedback. Practitioners will complete FidQ weekly after each session, evaluating their use of intervention components, pedagogical tools, client engagement, and responsiveness. The number of items varies depending on the components addressed each week. There are 21 predefined SPARCK components, and when one is selected, the practitioner is prompted to indicate the pedagogical tools utilized (i.e., verbal communication, demonstration, role play). At a minimum, ten items are answered weekly, including components used, pedagogical tools employed, home assignment given, and whether goals for the intervention were set or modified. When closing a case, an additional ten items are administered, covering changes in family’s goals, supervision the practitioner received, and a matrix evaluating family’s mastery of the various SPARCK components. Some further information can be found in the protocol for the intervention development [19] as well as the RCT protocol [21].

In addition to fidelity, the implementation outcomes that will be tested are acceptability, appropriateness, and feasibility [5, 35]. These will be measured using the 4-item scales Acceptability of Intervention Measure (AIM), Intervention Appropriateness Measure (IAM), and Feasibility of Intervention Measure (FIM) [48]. AIM, IAM, and FIM are measured on a 5-point Likert scale from 1 (completely disagree) to 5 (completely agree). Hence, higher scores indicate greater acceptability, appropriateness, and feasibility respectively.

#### Implementation determinants

Hypotheses H1, H2, and H4 will be tested using measures of implementation determinants. The 10-item version of Organizational Readiness for Implementing Change (ORIC) will be used to determine a collective level of organizational readiness for change [37]. The scale measures how well employees at an organization feel they can implement the change in processes required by a proposed intervention and the degree to which they are likely to initiate change, exert greater effort, exhibit greater persistence, and display more cooperative behavior. The measure consists of two subscales labeled Change Commitment (5 items) and Change Efficacy (5 items). Items are measured on a 5-point Likert scale from 1 (disagree) to 5 (agree). Sum score is calculated across all items. Lower scores represent less organizational readiness for implementing change; higher scores represent a more favorable organizational readiness for implementing change.

To measure other implementation determinants, we will use Implementation Determinants Measure (IDM). It consists of 47 items assessing factors that may hinder or foster successful implementation of an intervention into a given context. The measure is based on a 40-item scale, which was partly founded on CFIR [30] and partly on qualitative interviews with people involved in implementing mental health promotion programs in schools and kindergartens in Norway [32–34]. For the present study, IDM was adapted and customized to fit SPARCK and the municipal services in Norway, as well as the current study’s target problem areas. The measure includes seven items not included in the 40-item version. The adaptations and additional items cover findings gained from the semi-structured interviews with 14 SPARCK practitioners and 15 municipal leaders who used SPARCK in the two test cycles [19, 27]. Additionally, they cover items from pilot tests of the adapted version among relevant municipal practitioners and leaders. Three of the seven additional items came about after splitting the original items. Item 1a is “*The training in the innovation was of good quality*” and 1b “*The supervision of the innovation practitioners is carried out often enough*.” The original item combined training and supervision and quality and frequency in one item, which did not fit our intervention. Item 13 is split into three items, from the original one to cover three distinct target problem areas (prevention of development of anxiety, depression, and behavioral problems). As an example, item 13a reads “*There is a need for innovations that prevent the development of anxiety in children in Norwegian municipalities today*.” Pilot testing revealed a missing area in the measure’s content, leading to the addition of the following item to address it: 41 “*The innovation is appealing to our collaborating partners (i.e., other municipal services, out-patient clinics)*.” The pilot test and results from the prior qualitative implementation study [27] led us to add three more items, namely item 42 “*The ways of working with the innovation is compatible with existing clinical practice*,” item 43 “*We have the needed resources to work with the new innovation (staff, money, time, supplies, *etc*.)*,” and item 44 “*The innovation can easily be adapted to fit each family’s problem area and needs*.” Importantly, we made an effort to reflect the updated CFIR 2.0 [31] and an abbreviated measure of the CFIR-based Barrier Buster Tool when modifying and adapting it. The latter, developed by the CFIR Research Team in Ann Arbor, MI (based on unpublished work), assesses the constructs most reported to be associated with implementation outcomes. The final 47-item measure covers the CFIR domains innovation (14 items), outer setting (4 items), inner setting (8 items), individuals (8 items), and implementation process (13 items). Together, these comprise several sub-constructs (i.e., innovation relative advantage, innovation complexity, individuals’ attitudes, skills and motivation, external pressure, tension for change, implementation climate, available resources). Items are measured on a 7-point Likert scale from 1 (strongly disagree) to 7 (strongly agree). After reversing negatively phrased questions, a sum score will be calculated across all items. Lower scores indicate less positive environment for successful implementation; higher scores indicate more positive environment for successful implementation. In addition, one open ended question is included (“*Can you think of other factors that may affect the effectiveness or implementation of the intervention (positively or negatively)?*”). After the study period, the IDM will also be used to detect barriers within and across organizations and municipalities, to guide selection and/or tailoring of relevant implementation strategies if SPARCK is implemented and scaled up. Subsequently, data from the IDM will be used in a future study to identify and understand relations between implementation strategies and mechanisms of change [49].

#### Implementation strategies

Implementation strategies used during the clinical testing of SPARCK will be specified and reported throughout the study period (see Additional file 1 for a list of initiated and planned implementation strategies), along with reporting of and justification for tailoring of strategies. Strategies will be named and defined to align with the Expert Recommendations for Implementing Change (ERIC) compilation [25] and Proctor et al.’s [39] recommendations for specifying and reporting implementation strategies. Choice and adaptation of implementation strategies are based on NUBU’s implementation infrastructure and experience with implementing other EBIs [45], relevant implementation theories, models, and frameworks [26, 30, 31, 38], as well as data from the contextual analysis of barriers and facilitators to implementation in the development and optimization phase of the SPARCK project [27]. After study completion—and if SPARCK will be implemented into regular practice, there will be a follow-up study in which stakeholders will be asked to aid in choice and tailoring of appropriate implementation strategies to address barriers and facilitators, as well as investigation of implementation mechanisms [49].

### Data analysis

In implementation science, it is well-established that the contexts in which EBIs are implemented are inherently multilevel and interconnected [18]. Despite the recognized complexity, multilevel methods remain underutilized in implementation studies [50]. In our study, there are hierarchical structures that need to be either accounted for or modeled. For instance, families are nested within practitioners and leaders who in turn are nested within municipalities, and for some of the outcomes multiple responses over time are nested within individuals (i.e., practitioners and leaders). Given this longitudinal and hierarchical structure of our data, we will use multilevel models (MLM). In general, we will conduct multilevel regression analyses to investigate relations between predictors and outcome variables. However, despite the multilevel structure of the data, we do not expect nesting at all possible levels to be relevant. Thus, investigation of intraclass correlation coefficients (ICC) and design effects will guide our decisions regarding modeling [51].

More specifically, in H1, which concerns implementation determinants as predictors of implementation outcomes, we will regress implementation outcomes at T2 on implementation determinants at T1. In essence, this means a potential two-level model (practitioners/leaders are nested within municipalities). To investigate H2, concerning a relation between implementation determinants and parent reported clinical outcomes, estimates of change from pre to post or pre to follow-up [21] in child symptoms will be regressed upon either a mean-variable of practitioner/leader reported implementation determinants at T1, T2, and T3, or practitioner/leader reported implementation outcomes as a time-varying covariate (predictor). The same strategy will be used to investigate H3, only with implementation outcomes as predictor. In these analyses, there are potentially three levels (families nested within practitioner/leader which are nested in municipalities). Importantly though, we aim towards a parsimonious model in which complexity in the model is introduced only if the first steps in the model suggest it is necessary (e.g., through high ICC and design effects). To investigate H4, concerning a positive development in ratings of implementation determinants and outcomes over time, a multilevel growth curve model with three levels (repeated measures [level 1], nested within practitioners/leaders [level 2], nested within municipalities [level 3]) could be a solution given the nesting in our data. However, as we have a small sample size and data over time is gathered with one year in between, we will consider a less complex model [52]. Across all analyses, model building will be conducted in a stepwise fashion and improvement will be assessed for each step [53, 54] through model comparisons using -2LL, AIC, and BIC. Thus, maximum likelihood estimation is used for model comparisons and restricted maximum likelihood for final parameter estimates. Missing data will be handled using full information maximum likelihood, in which all available data in the model contribute to the parameters in the final model [55]. Although we have not specified specific mediator and moderator analyses in the present protocol study, we will explore potential theoretically and empirically relevant mediators and moderators in cross-level mediation and interactions models. Finally, we will explore differences between municipalities involved in the optimization process and those not involved, to assess whether co-creation is associated with implementation outcomes.

## Discussion

This protocol outlines the rationale and design of an implementation study of SPARCK into multiple sites in Norwegian municipalities. SPARCK is a carefully co-created and optimized parent training intervention for prevention of child mental health problems. In addition to evaluating the clinical effect of SPARCK in an RCT, we aim to investigate the importance of various implementation factors, as effectiveness and implementation research are stronger when conducted in tandem [14]. A dual focus on effectiveness and implementation represents a potential to speed up the uptake of an intervention, more effective implementation strategies, and more useful information for both researchers and decision-makers [43, 44].

How to improve effectiveness of interventions targeting child mental health problems, and how to optimize their implementation and sustained use constitute major challenges, both applied and scientific, within prevention and implementation research. If children and families do not have access to EBIs they could have profited from, the implications may be severe at both personal and societal levels and include prolonged suffering for the families. The presented study may generate much-needed knowledge on how implementation determinants impact clinical outcomes, as well as specific implementation outcomes. An additional aim of the study is to explore and report on implementation strategies that are needed to promote sustainable long-term outcomes of parent training interventions for prevention of child mental health problems in Norway and beyond.

Scientifically, the effectiveness study in this project may contribute to narrowing the knowledge gap on the effect of parent training interventions for child internalizing and externalizing symptoms [21]. The implementation study may contribute to the implementation science field with knowledge on how contextual barriers can affect both clinical effectiveness and specific implementation outcomes that are important in the initial phase of implementation. This knowledge is essential when choosing, tailoring, and deploying implementation strategies [6, 25, 26] to address barriers, and ultimately to promote success and sustainment when implementing EBIs beyond the scope of a research study and into the real world [18].

## Supplementary Information

Additional file 1: List of planned and initiated implementation strategies.

Additional file 2: TIDieR checklist.

## Data Availability

A comprehensive plan outlining the procedures for the collection, handling, and storage of data has received approval from SIKT. To ensure the security and privacy of sensitive information, we use the University of Oslo’s Services for Sensitive Data (TSD) [58] for all data storage and analysis. We use Nettskjema [59], developed and operated by the University Information Technology Center, a TSD-integrated survey solution for collecting sensitive data. All study information, including participant identification keys and raw data, will be securely stored at TSD. Access to this information will be restricted to authorized personnel. We will implement measures to protect data confidentiality and integrity, including encryption of all data transfers and regular backups to safeguard against data loss. In the event of any breaches or unauthorized access, we will follow established protocols for reporting and responding to such incidents. The datasets generated in the current project are not publicly available due to the General Data Protection Regulation (GDPR) and Norwegian regulations but can be made available upon request.

## Abbreviations

AIM: Acceptability of Intervention Measure
CFIR: Consolidated Framework for Implementation Research
EBIs: Evidence-based interventions
ERIC: Expert Recommendations for Implementing Change compilation
FidQ: Fidelity Questionnaire
FIM: Feasibility of Intervention Measure
GDPR: General Data Protection Regulation
IAM: Intervention Appropriateness Measure
IDEAS: Innovate, Develop, Evaluate, Adapt, Scale Impact Framework
IDM: Implementation Determinant Measure
IRLM: Implementation Research Logic Model
MOST: Multiphase Optimization Strategy
NUBU: Norwegian Center for Child Behavioral Development
ORIC: Organizational Readiness for Implementing Change
PI: Principal investigator
PMTO: Parent Management Training—Oregon model
REK: Regional Committee for Medical Research Ethics in Norway
RCT: Randomized controlled trial
SCED: Single case experimental designs
SIKT: Norwegian Agency for Shared Services in Education and Research
SPARCK: Supportive Parents—Coping Kids
T1: Time 1
T2: Time 2
T3: Time 3
TIDieR: Template for Intervention Description and Replication checklist and guide statement
TSD: University of Oslo’s Services for Sensitive Data
UCD: User-centered design
