# Supplementary material for: Implementation of a parent training intervention (SPARCK) to prevent childhood mental health problems: study protocol for a pragmatic implementation trial in Norwegian municipalities
Source: Trials. 2024 Dec 21;25:846. doi: 10.1186/s13063-024-08704-7 (PMC11663316; doi:10.1186/s13063-024-08704-7)
Supplement: Supplementary file 1 — Additional file 1: List of planned and initiated implementation strategies. [file 13063_2024_8704_MOESM1_ESM.docx]

**Additional file 1, list of initiated and planned** **implementation strategies**

After two optimization test cycles and co-creation of SPARCK, we initiated and planned the following 26 discrete and multifaceted implementation strategies. The time span for the strategies ranges from fall 2022 to fall 2027, which includes preparation, initiation, and completion of the current study, as well as practical implementation of SPARCK in the regular services, given positive effects in the RCT (see protocol for RCT study [1]). The strategies are named, defined, and specified according to Proctor et al.’s recommendations (2). They are defined to align with the Expert Recommendations for Implementing Change (ERIC) compilation (3), and clustered according to Waltz et al.’s categories (4). The conceptual action targets are operationalized to address potential barriers to SPARCK adoption, implementation, and sustainment. The supposed affected implementation outcomes correspond with Proctor et al.’s Conceptual Framework for Implementation Outcomes (5).

The strategies are selected, developed or adapted based on experiences from co-creating the SPARCK intervention (see protocol for intervention development [6]), data from the parallel contextual analysis (7), the current study’s aims and hypotheses, experiences from implementing other EBIs and alignment with a pre-existing implementation infrastructure (8), as well as to correspond with relevant theory and existing knowledge in the field (2-5, 9-13).

| **Strategy** | | **Definition** | **Actor** | **Action** | **Action target** | **Temporality** | **Dose** | **Imp. outcome affected** | **Justification** |  |  |
| --- | --- | --- | --- | --- | --- | --- | --- | --- | --- | --- | --- |
| **Use evaluative and iterative strategies (n = 5)** | | | | | | | | | |  |  |
| 1 | *Assess for readiness and identify barriers and facilitators* | *Assess organizational readiness to implement the new intervention, as well as identification of determinants that may influence the implementation effort.* | Research team (RT) | Administer web-based contextually adapted versions of ORIC and IDM. | (*Individual target*) SPARCK practitioners (SP) and municipal leaders (ML).  (*Conceptual target*)  Perceived organizational readiness and identification of contextual barriers and facilitators. | Three time points during study; T1, T2, and T3. | Approx. 15-20 minutes. | Adoption, appropriateness, feasibility, acceptability, penetration, sustainability. | (*Theoretical*)  (9-12)  (*Empirical*) Prior experience with implementing EBIs in Norwegian municipalities and current study’s aims and hypotheses. |  |  |
| 2 | *Conduct local needs assessment* | *Collect and analyze data related to the need for the innovation.* |  | *Multifaceted strategy that occurs in several domains and at several levels.* | (*Conceptual target*) Gain access to knowledge and information from relevant stakeholders. Characterize and identify problem areas and local needs and expectations. Build consensus and increase readiness. |  |  | Adoption, appropriateness feasibility, sustainability. | (*Theoretical*)  (4, 9, 10, 14)  (*Empirical*)  data and experiences from SPARCK optimization study^a^ (6, 7). |  |  |
|  |  |  | RT | 2a) analyze and use data from SPARCK optimization study^a^ when choosing and adapting implementation strategies. | (*Individual target*) SP and ML in SPARCK optimization study^a^. | Data collected prior to current study. |  |  |  |  |  |
|  |  |  | RT, SPARCK developers (SD) | 2b) hold municipality workshops^b^. | (*Individual target*)  SP, SS, ML, and other municipal stakeholders, national implementation team (NIT)^c^ | After municipalities were recruited, and prior to training of SP. | Each municipality attended one workshop (2,5 hours). |  |  |  |  |
|  |  |  | RT, SD | 2c) conduct The Lab seminars^d^ | (*Individual target*)  SP in the Lab^e^ | During study, after one year. | Seminars last 1-2 days. |  |  |  |  |
|  |  |  | RT | 2d) perform a modified Delphi study^f^ and/or semi-structured interviews. | (*Individual target*)  SP, SS, ML, other relevant municipal stakeholders, implementation experts. | After current study. | Two or three-rounds, each round will take 30-45 min. If interviews, 45-60 min. |  |  |  |  |
| 3 | *Develop a formal implementation blueprint* | *Develop a formal implementation blueprint that includes all goals and strategies.*  *The blueprint should include the following: 1) aim/purpose of the implementation.*  *2) scope of the change (e.g., what organizational units are affected), 3) timeframe*  *and milestones, and 4) appropriate performance/progress measures. Use and*  *update this plan to guide the implementation effort over time.* | RT, SD, NIT^c^ | A SPARCK implementation plan^g^ is initiated. | (*Individual target*)  SPARCK supervisors (SS), SP, ML  (*Conceptual target*)  Ensure commitment and feedback from stakeholders, as well as allocation of necessary local infrastructure and resources. Can be used as a historical record of the implementation process at each site, and a documentation of balance between fidelity and adaptability. | Initiated prior to study, used, and updated throughout study by RT and SD. Tailored to each site after study by SD and NIT^c^, and continually updated. | As needed throughout study, with most efforts after study in workshops and meetings with relevant stakeholders. | Fidelity, sustainability, appropriateness, feasibility, cost. | (*Theoretical*)  (3)  (*Empirical*)  Prior experience with implementing EBIs in Norwegian municipalities (8). |  |  |
| 4 | *Develop and organize quality monitoring systems* | *Develop and organize systems and procedures that monitor clinical processes*  *and/or outcomes for the purpose of quality assurance and improvement.* | SD and staff at NUBU | Inclusion of SPARCK into existing electronic quality monitoring system^h^. | (*Individual target*)  SP, SS, ML  (*Conceptual target*)  For users and municipality stakeholders, it is important for transparency and creating ownership. When clinical and implementation outcomes are available, it is a source for quality assurance and fidelity checking. | During study, SPARCK manuals and materials^i^, and information about the study will be available for SP and SS. After study, further quality monitoring functionality can be added. | As needed by SD and administrative staff. After study, SP and SS can populate the system with case, training, and supervision data, and extract reports as needed. | Fidelity, penetration, cost, sustainability. | (*Pragmatic*)  The system has been in place for over ten years and all the municipalities are familiar with it. Users are used to it expanding and gaining functionality for other EBIs. |  |  |
| 5 | *Obtain and use patients/ consumers and family feedback* | *Develop strategies to increase patient/consumer and family feedback on the*  *implementation effort* | RT, SP | Administration of a client satisfaction questionnaire in the RCT. Items concern quality of the intervention, and whether it matched their needs and expectations. | (*Individual target*)  Parents of target child.  (*Conceptual target*)  Being centered on the clients and taking their feedback on aspects of the intervention and implementation effort into account, may improve organizations’ likelihood to implement change effectively. | During RCT T3 (post intervention)  After study, client feedback can be obtained in sessions and relayed to supervisors. | Each questionnaire takes around two minutes to fill out. | Appropriateness, adoption. | (*Theoretical*)  (9, 10)  (*Empirical*)  (1) |  |  |
| **Provide interactive assistance (n = 2)** | | | | | | | | | |  |  |
| 6 | *Facilitation* | *A process of interactive problem-solving and support that occurs in a context of a*  *recognized need for improvement and a supportive interpersonal relationship.* |  | *Multifaceted strategy* | (*Conceptual target*)  According to the PARIHS framework, facilitation is the core and active ingredient  to address barriers and leverage facilitators to increase likelihood for successful implementation. |  |  | Feasibility, appropriateness, sustainability. | (*Theoretical*)  (15, 16)  (*Pragmatic*) NUBU has a pre-existing implementation infrastructure^j^ (8), which the current intervention can be integrated into. Facilitation activities occur via implementation teams at national, regional, and local levels^j^. |  |  |
|  |  |  | RT, SD | 6a) SPARCK research and development core group^k^ meetings. | (*Individual target*)  RT and SD. | Weekly/ biweekly throughout study period. | One-hour meetings weekly/ biweekly. |  |  |  |  |
|  |  |  | RT | 6b) research coordinators are in contact with study participants to support, inform, nudge, and problem solve practical issues and uncertainties regarding study. | (*Individual target*)  SS, SP, and study participants. | Regular contact as needed throughout the study period, concentrated around data collection points. | As needed throughout study period. |  |  |  |  |
|  |  |  | RT, SD, NIT^c^ | 6c) NIT^c^ and SS have established formal and informal interpersonal relationships with municipalities, making them particularly suited for facilitating context specific issues. | (*Individual target*)  SS, SP, ML, and other municipal stakeholders. | NIT^c^ and SS are available for contact and facilitation as needed throughout study. After study, SPARCK can easily be included within this implementation infrastructure^j^. | As needed throughout study period. After study, more formalized points of contact and facilitation needs will be established. |  |  |  |  |
| 7 | *Provide clinical supervision* | *Provide clinicians with ongoing supervision focusing on the innovation. Provide*  *training for clinical supervisors who will supervise clinicians who provide the*  *innovation.* |  | *Multifaceted strategy* | (*Conceptual target*)  Provision of ongoing support and consultation after initial training is crucial to ensure fidelity, above and beyond type of training method.  Supervision is important for monitoring clinical processes  and outcomes. |  |  | Fidelity, sustainability. | (*Theoretical*)  (17)  (*Empirical*)  Based on feedback in SPARCK optimization study^a^, frequency of supervision was reduced (6, 7).   (*Pragmatic*) SS and SP have an established inter-relationship through the implementation infrastructure^j^ of other EBIs, which include supervision. |  |  |
|  |  |  | SD | 7a) SD train SS in the intervention, with a particular focus on supervision issues related to SPARCK. | (*Individual target*)  SS | Fall 2022, prior to training of SP. Themes relating to SPARCK will also be discussed in regular NIT^c^ meetings, which SS attend. | Two full days. |  |  |  |  |
|  |  |  | SS | 7b) SS conduct ongoing clinical supervision in groups with SP from municipalities in their region. During SP first two cases, supervision is more frequent, considered part of the training and labelled “training supervision”. From case three and onwards, supervision is less frequent and labelled “maintenance supervision”. | (*Individual target*)  SP | During SP first year with SPARCK (first two cases), weekly supervision. Thereafter, biweekly supervision. | Each SP gets approx. 30 min. in supervision session for their case. They listen in on group members’ case supervision. Groups have 2-3 SP, each supervision session lasts between 1-1,5 hours. |  |  |  |  |
| **Adapt and tailor to context** **(n = 2)** | | | | | | | | | |  |  |
| 8 | *Tailor strategies* | *Tailor the implementation strategies to address barriers and leverage facilitators*  *that were identified through earlier data collection.* |  | *Multifaceted strategy* | (*Conceptual target*) Identifying and targeting barriers to implementation is essential. Due to heterogeneity in service sites, a strategy may be successful in one context, but not in others, therefore tailoring is important. |  |  | Adoption, acceptability, appropriateness, feasibility, fidelity, sustainability. | (*Theoretical*)  (9, 14, 18)  (*Empirical*)  Data and experiences from SPARCK optimization study^a^ (6, 7) |  |  |
|  |  |  | RT, SD | 8a) the SPARCK optimization study^a^ identified various barriers and facilitators and adaptations were made based on these. Fidelity will be assessed throughout study, and further tailoring to address contextual barriers will be documented throughout study. | (*Individual target*)  Depends on strategies that will be tailored. | During the study, the tailoring of implementation strategies will be documented and reported. | As appropriate during study, if it is deemed that tailoring is necessary. |  |  |  |  |
|  |  |  | RT, SD | 8b) after study, implementation strategies will be tailored in follow up study, to reflect current and earlier study findings. | (*Individual target*) ML and other relevant municipal stakeholders, SS, SP, NIT^c^, and implementation experts. | After current study, a modified Delphi study^f^ will commence and/or semi-structured interviews. | Two or three rounds, each round will take 30-45 min. If interviews, 45-60 min. per person. |  |  |  |  |
| 9 | *Promote adaptability* | *Identify the ways a clinical innovation can be tailored to meet local needs and*  *clarify which elements of the innovation must be maintained to preserve fidelity.* | RT, SC | SPARCK’s theory of change is defined and specified, and core elements are defined and described in materials^i^. If the current study warrants improvements to intervention content and materials, it will be changed prior to implementation in real-world settings. Tailoring of implementation strategies to local needs will be made together with relevant stakeholders after current study. | (*Individual target*)  SS, SP, ML, and other relevant stakeholders  (*Conceptual target*) Fidelity represents internal validity while adaptability represents external validity. Adaptability improves fit between intervention and context. Promoting adaptability while preserving fidelity can pose a dilemma but needs to be addressed and balanced to the needs of the end users and stakeholders. | During study SP will receive clinical supervision by intervention experts and fidelity will be measured and tracked closely and investigated in relation to clinical outcomes.  After current study, a modified Delphi study^f^ will commence and/or semi-structured interviews. | Two or three round Delphi survey, each round will take 30-45 min. If interviews, 45-60 min. per person. | Fidelity, appropriateness, feasibility, and sustainability. | (*Theoretical*)  (9, 10, 19, 20)  *(Empirical)* Through cyclical tests of change, we have tested out different versions of the SPARCK intervention, based on quantitative and quantitative data and feedback from relevant stakeholders and participants. |  |  |
| **Develop stakeholder interrelationships (n = 7)** | | | | | | | | | |  |  |
| 10 | *Build a coalition* | *Recruit and cultivate relationships with partners in the implementation effort.* |  | *Multifaceted strategy* | (*Conceptual target*) Promotes relational connections and supports preexisting work infrastructure. Fosters cooperation and alliance among formal and informal stakeholders at multiple levels. |  |  | Fidelity, appropriateness, feasibility, and sustainability. | (*Theoretical*)  (9, 10) |  |  |
|  |  |  | RT, SD | 10a) Researchers and clinicians in the NUBU core group^k^ work closely together in the current trial. | (*Individual target*)  RT, SD | Weekly/ biweekly meetings throughout study period. Additional meetings if needed. | One-hour meetings weekly/ biweekly, depending on need. |  | (*Empirical*)  Positive experiences from SPARCK optimization study^a^.  (*Pragmatic*) Updates and problem solving should occur regularly. RT and SD are employed in same organization, making weekly/biweekly meetings convenient. |  |  |
|  |  |  | NIT^c^ | 10b) The regional members of NIT^c^ have formal links to stakeholders in municipalities where SPARCK takes place. NIT members will be SS for their region’s SP. NIT is an important partner for the current study and will central if SPARCK will be implemented in routine practice. | (*Individual target*)  SS, ML, and other municipal stakeholders. | NIT meets five to six times a year, in addition to regular correspondence as needed. | b) Two-day meetings, five to six times a year. SPARCK related themes are given priority as suited, usually 1-2 hours each meeting. |  | (*Empirical*) Positive experiences with partnering with NIT from other studies (8).  (*Pragmatic*) NIT’s meeting structure is established, making it a suitable arena for coalition building without adding meeting points. |  |  |
| 11 | *Capture and share local knowledge* | *Capture local knowledge from implementation sites on how implementers and*  *clinicians made something work in their setting and then share it with other sites.* |  | *Multifaceted strategy* | (*Conceptual target*) Give access to knowledge and information. Increase understanding, as implementation issues are operationalized and contextualized to similar practice settings. Promote information sharing and peer learning. Cultivate a feeling of togetherness. Promotes helping behavior and expansion of one’s social networks. Can foster later collaborative learning environment between sites and municipalities, which can improve implementation of SPARCK. |  |  | Adoption, acceptability, appropriateness, feasibility, sustainability. | (*Theoretical*)  (3, 9, 10)  (*Empirical*) Awareness-building, knowledge-acquisition  (*Pragmatic*) Low cost/ resource-effective way of knowledge sharing. |  |  |
|  |  |  | SS, SP | 11a) SP can share information with SS and other SP in supervision groups. Most of the time will concern the clinical case, however, 5-10 min. each session can be allocated running issues and problem solving. | (*Individual target*)  SP, SS | Weekly during SP first year with SPARCK, biweekly during second year. | Approx. 5-10 min. each session. |  |  |  |  |
|  |  |  | SD, SS | 11b) SS can capture and share information with SD in supervision-of-supervisor groups, who again can share with RT. Most of the time will concern clinical and process issues of the SP in SS groups, however, 5-10 min. can be allocated running issues and problem solving. | (*Individual target*)  SD, SS | Approx. biweekly, depending on need. | Approx. 5-10 min. each session. |  |  |  |  |
|  |  |  | RT | 11c) Some RT members are in contact with study participants and can capture local knowledge. | (*Individual target*)  SP, ML | Available for contact during study period. |  |  |  |  |  |
|  |  |  | RT, SD | 11d) Study updates and information are shared with study participants and stakeholders in information letters distributed via e-mail. More formal documents. i.e., suggested responses to specific barriers can be shared on the electronic quality monitoring system^h^. | (*Individual target*)  SS, SP, ML | Information e-mails will be distributed approx. biweekly. Formal documents will be uploaded to electronic system^h^ as needed. |  |  |  |  |  |
|  |  |  | RT, SD | 11e) The Lab seminar^d^  to capture the first wave sites' experiences with making SPARCK work in their settings.  Mix of information sharing, group tasks and focus group interview. | (*Individual target*)  SP in the Lab^e^ | After approx. one year | 1-2 days |  |  |  |  |
|  |  |  | RT | 11f) Results from SPARCK optimization study^a^ will be shared in scientific journals and conferences. |  | Journal articles and conference presentations will occur occasionally during study period. |  |  |  |  |  |
| 12 | *Conduct local consensus discussions* | *Include local providers and other stakeholders in discussions that address whether*  *the chosen problem is important and whether the clinical innovation to address it*  *is appropriate.* |  | *Multifaceted strategy* | *(Conceptual target*) Give access to knowledge and information to providers and stakeholders. Characterize and identify problem area. Build consensus and increase readiness. |  |  | Adoption, appropriateness, feasibility. | (*Theoretical*)  (9, 10)  (*Empirical*) Awareness-building, knowledge-acquisition. |  |  |
|  |  |  | NIT^c^ | 12a) Informational meetings with eligible municipalities, which included discussions of addressing target group, problem area, needs and information about SPARCK intervention. | (*Individual target*)  SP, SS, ML, and other municipal stakeholders. | When municipalities were recruited. | Varied in dosage, approx. 1-2-hour meetings at each site. |  |  |  |  |
|  |  |  | RT, SD | 12b) Municipality workshops^b^. | (*Individual target*) SP, SS, ML, and other municipal stakeholders. | After municipalities were recruited, and prior to training of SP. | Each workshop lasted 2,5 hours. |  |  |  |  |
|  |  |  | RT, SD | 12c) The Lab seminars^d^. | (*Individual target*)  SP in the Lab^e^ | After approx. one year. | 1-2 days. |  |  |  |  |
|  |  |  | RT | 12d) Perform a modified Delphi study^f^ and/or semi-structured interviews. | *(Individual target)*  SP, SS, ML, other relevant municipal stakeholders, implementation experts | After current study. | Two or three rounds, each round taking 30-45 min. If interviews, 45-60 min. per person. |  |  |  |  |
| 13 | *Identify and prepare champions* | *Identify and prepare individuals who dedicate themselves to supporting, marketing,*  *and driving through an implementation, overcoming indifference or resistance that*  *the intervention may provoke in an organization.* | NIT^c^, SD | During the SPARCK optimization study^a^, early adopters were identified and recruited to be first wave SP (the Lab^e^). These SP are prepared and motivated for the role as champions. | (*Individual target*)  SP in the Lab^e^  *(Conceptual target)* Champions can advocate for SPARCK within their organization, motivate staff, inform, and build relationships with leaders and other stakeholders. In addition, they can troubleshoot problems that emerge during implementation with NIT and SD. | Identified prior to current study. Prepared in add-on training following new SP training, and during the Lab seminars^d^. |  | Adoption, appropriateness, feasibility, sustainability. | *(Theoretical)*  (3, 9, 21)  (*Empirical and pragmatic*)  The municipalities are part of a pre-existing tiered implementation infrastructure^j^. Identifying possible champions as members of local implementation teams has been an important source for success in the past (8). |  |  |
| 14 | *Obtain formal commitments* | *Obtain written commitments from key partners that state what they will do to*  *implement the innovation.* |  | *Multifaceted strategy* | (*Conceptual target*)  Ensure commitment from stakeholders, as well as allocation of necessary local resources. |  |  | Adoption, sustainability. | *(Empirical)* (8)  (*Pragmatic*)  Municipalities NUBU work with are used to sign and commit to formal commitments. |  |  |
|  |  |  | RT, SD, NIT^c^ | 14a) The municipalities commit to participation in study via formal contracts. | (*Individual target*)  Relevant municipal stakeholders. | Prior to study. |  |  |  |  |  |
|  |  |  | RT, SD | 14b) in municipality workshops^b^, all recruited municipalities were asked to start making a simple study implementation plan^g^ and send to us after workshop. | (*Individual target*)  ML, SP, and other relevant municipal stakeholders. | Prior to study, after municipality workshops^b^. |  |  |  |  |  |
|  |  |  | SD, NIT^c^ | 14c) written agreements will be obtained that will enable current and future SP to fulfill and commit to training and supervision, adhere to quality assurance procedures, and leaders to plan for future. Supplements will be detailed in the formal implementation blueprint/plan. | (*Individual target*)  ML and other relevant municipal stakeholders. | After study. | As needed. Will normally be obtained after one or two informational meetings with decision makers and relevant stakeholders. |  |  |  |  |
| 15 | *Organize clinician implementation team meetings* | *Develop and support teams of clinicians who are implementing the innovation*  *and give them protected time to reflect on the implementation effort, share*  *lessons learned and support one another’s learning.* | SD, NIT^c^ | The implementation team infrastructure^j^ will take over facilitation support after the current study. | (*Individual target*)  SS, SP, other relevant municipal stakeholders  (*Conceptual target*) Give access to knowledge and practical information. Increase understanding, as implementation issues are operationalized and contextualized. Promotes information sharing and peer learning. Cultivate a feeling of togetherness. Promotes helping behavior and expansion of one’s social networks. | After current study, if decision to implement into practice is formed.  Details regarding frequency, type and temporality of contact will be formalized in implementation blueprint in collaboration with relevant stakeholders. | To be decided. | Fidelity, appropriateness, sustainability. | *(Empirical)* (8)  (*Pragmatic*)  Municipalities are used to the implementation infrastructure^j^, and the tasks associated with it.   Low cost/ resource-effective way of knowledge sharing. |  |  |
| 16 | *Use advisory boards and workgroups* | *Create and engage a formal group of multiple kinds of stakeholders to provide input and advice on implementation efforts and to elicit recommendations for improvements.* |  | *Multifaceted strategy* | (*Conceptual target*) Ensure commitment from stakeholders. Fosters cooperation and alliance among formal and informal stakeholders at multiple levels. |  |  | Acceptability, appropriateness, feasibility, sustainability. | *(Empirical)* Positive experiences during SPARCK optimization study^a^, as well as experiences with the existing implementation infrastructure^j^. |  |  |
|  |  |  | SD | 16a) members of NIT^c^ (internal stakeholders) are trained in SPARCK intervention and are supervisors to SP and responsible for follow-up of municipalities in their region. As they are experts in municipal implementation of other EBIs, they are an important source of input and feedback. | (*Individual target*)  NIT | NIT meet five to six times a year, in addition to regular correspondence as needed. | Two-day meetings, five to six times a year. SPARCK related themes are given priority as suited, usually 1-2 hours each meeting. |  |  |  |  |
|  |  |  |  | 16b) the Lab^e^ (external stakeholders) were engaged before, throughout and after current study, and will be an important advisory group for feedback and improvements. | (*Individual target*)  SP in the Lab^e^ | A seminar^d^ one year into current study. To be decided after study. | 1–2-day seminar. To be decided after study. |  |  |  |  |
|  |  |  | RT, SD | 16c) A formal group of  user representatives have been engaged to give feedback and input on intervention, study, and information materials. | (*Individual target*)  Representatives from a municipality and a national interest group on mental health issues. | Revision of study protocol and materials prior to study. Informational meetings during study. | One meeting a year during study or as needed. |  |  |  |  |
| **Train and educate stakeholders (n = 7)** | | | | | | | | | |  |  |
| 17 | *Conduct educational meetings* | *Hold meetings targeted toward different stakeholder groups (e.g., providers,*  *administrators, other organizational stakeholders, and community, patient/ consumer, and family stakeholders) to teach them about the clinical innovation.* | RT, SD | Several educational meetings have and will be held, for various stakeholders, targeted to their needs.   Information and workshop^b^ meetings were held digitally, directed at all recruited municipalities. | (*Individual target*)  potential new SP, SP in the Lab^e^,  ML and other relevant municipal stakeholders, SS  (*Conceptual target*) Give access to knowledge and practical information to various stakeholders. | Fall 2022, after municipalities were recruited, and prior to training of SP.   Other educational meetings will be held as needed. | Five meetings were held, each lasting around 2,5 hours. | Acceptability, appropriateness, feasibility. | (*Theoretical*) (3)  (*Empirical*) Awareness-building, knowledge-acquisition. |  |  |
| 18 | *Conduct ongoing training* | *Plan for and conduct training in the clinical innovation in an ongoing way.* |  | *Multifaceted strategy.*  Group based training in the SPARCK intervention, for three different groups. | (*Conceptual target*)  Knowledge acquisition, which will enable clinicians to perform the clinical intervention with fidelity. |  |  | Fidelity, acceptability, cost. | (*Theoretical*) (3)  (*Pragmatic*)  All SP and SS are experienced clinicians who are used to training others and attend training in other EBIs. Conducting three separate training set ups was cost efficient as well as tailored to the practitioners’ experience with SPARCK and role in current study. |  |  |
|  |  |  | SD | 18a) add-on training in the optimized version of SPARCK, based on their preexisting knowledge and experience with using the intervention. | (*Individual target*)  SP in the Lab^e^ | Prior to current study and one year into study. | One-day training prior to study, and half day training in after one year. |  |  |  |  |
|  |  |  | SD | 18b) training in clinical intervention and training in how to supervise SP in SPARCK. Part of training include having clients. | (*Individual target*)  SS | Fall 2022, as well as ongoing booster trainings at NIT^c^ meetings throughout study period. | Two-day training, as well as training sessions as needed. |  |  |  |  |
|  |  |  | SD | 18c) training in the clinical intervention. | (*Individual target*)  SP | Beginning of 2023. | Four-day training. |  |  |  |  |
| 19 | *Develop educational materials* | *Develop and format manuals, toolkits, and other supporting materials in ways that*  *make it easier for stakeholders to learn about the innovation and for clinicians to*  *learn how to deliver the clinical innovation.* | SD | Detailed SPARCK manuals and materials^i^ were developed and refined over years. | (*Individual target*)  SP, SS, clients  (*Conceptual target*)  Knowledge acquisition, which will enable clinicians to perform the clinical intervention with fidelity. | Prior to current study. | Development and refinement of materials went on between 2019-2023. | Fidelity, acceptability, sustainability. | (*Theoretical*) (3)  (*Empirical*)  SD are experienced in developing educational materials for new interventions as well as refinement of existing interventions, directed at both new and experienced clinicians as well as clients. |  |  |
| 20 | *Distribute educational materials* | *Distribute educational materials (including guidelines, manuals, and toolkits) in*  *person, by mail, and/or electronically.* | SD and NUBU staff | SP and SS have access to and use NUBU’s electronic quality monitoring system^h^, where all SPARCK manuals and materials^i^ are available. | (*Individual target*)  SP, SS, ML  (*Conceptual target*)  Ease of access. Give access to knowledge and practical information. | During study, SPARCK manuals and materials^i^ will be available for SP and SS. Other educational materials can be added after study period. | As needed during study. After study, SP, SS, and local coordinators can populate each municipality’s site with additional educational materials as needed. | Feasibility, cost, sustainability. | (*Pragmatic*)  All the  municipalities are familiar with and use NUBU’s electronic quality monitoring system^h^ and are used to it expanding and gaining functionality. |  |  |
| 21 | *Make training dynamic* | *Vary the information delivery methods to cater to different learning styles and*  *work contexts and shape the training in the innovation to be interactive.* | SD | Training is interactive and delivered using different pedagogical tools and methods, such as demonstrations, role play, breakout groups, engagement in problem solving, in addition to verbal teaching. Each training day, the separate themes are delivered by different SD. | (*Individual target*)  SP, SS  (*Conceptual target*)  Knowledge acquisition and increased understanding. Ensure commitment and engagement. | Prepared prior to study, performed during training. | Each theme in training sessions includes various dynamic delivery methods. | Acceptability, fidelity. | (*Theoretical*) (3)  (*Empirical*)  SD are experienced in teaching EBIs and counselling skills to others. |  |  |
| 22 | *Provide ongoing consultation* | *Provide ongoing consultation with one or more experts in the strategies used to*  *support implementing the innovation.* | SD | In current study labelled “supervision-of-supervisor”.  SD provide ongoing web-based consultation to groups of supervisors, both on clinical issues SS supervise SP on, as well as process and implementation issues. | (*Individual target*)  SS  (*Conceptual target*)  Provision of ongoing support and consultation after initial training is crucial to ensure fidelity, above and beyond type of training method. | Approx. biweekly, depending on need. | Each SS gets approx. 30 min. for their case. They listen in on group members’ supervision. Groups have 2-3 SP, each supervision session will last between 1-1,5 hours. | Fidelity, sustainability. | (*Theoretical*)  (17)  (*Pragmatic*) SD and SS have an established implementation infrastructure regarding supervision and are used to current set-up. |  |  |
| 23 | *Use train-the-trainer strategies* | *Train designated clinicians or organizations to train others in the clinical*  *Innovation.* | SD, SS, and SP in the Lab^e^ | SS are trained to supervise others and post study they are designated trainers and supervisors of SP in their region.   SP in the Lab^e^ are champions, have more experience in using SPARCK and were recruited deliberately as future candidates as SPARCK trainers and supervisors. | (*Individual target*)  Future SP. | Post study period. | To be decided. | Cost, penetration, sustainability. | (*Theoretical*)  (8)  (*Empirical and pragmatic*) Municipalities NUBU work with are used to the implementation infrastructure^j^ and the tasks associated with it.   Low cost/ resource-effective way of scale-up of intervention. |  |  |
| **Support clinicians (n = 2)** | | | | | | | | | |  |  |
| 24 | *Remind clinicians* | *Develop reminder systems designed to help clinicians to recall information and/or prompt them to use the clinical innovation.* |  | *Multifaceted strategy* | (*Conceptual target*) Give access to knowledge and information, increase understanding. |  |  | Adoption, fidelity. | (*Empirical*) Awareness-building, knowledge-acquisition  (*Pragmatic*) Low cost/ resource-effective way of knowledge sharing. |  |  |
|  |  |  | SS | 24a) SS can remind SP in supervision groups. | (*Individual target*)  SP | Weekly during SPs first year with SPARCK, biweekly during second year. | As needed. |  |  |  |  |
|  |  |  | SD | 24b) SD can remind and prompt SS in supervision-of-supervisor groups. | (*Individual target*)  SS | Approx. biweekly. | As needed. |  |  |  |  |
|  |  |  | RT | 24c) research coordinators will remind and be in contact with SPs as needed. | (*Individual target*)  SP | Throughout study period. | As needed. |  |  |  |  |
|  |  |  | RT, SD | 24d) RT and SD share updates and information with all SPs and ML in information letters distributed via e-mail. More formal documents can be shared on electronic quality monitoring system^h^. | (*Individual target*)  SP, ML | Information e-mails distributed approx. biweekly during study period. Formal documents are uploaded as needed. |  |  |  |  |  |
|  |  |  | RT, SD | 24e) All training materials^i^ and video recordings of training are available on electronic quality monitoring system^h^. | (*Individual target*)  SP, SS | Throughout study period. |  |  |  |  |  |
| 25 | *Facilitate relay of clinical data to providers* | *Provide as close to real-time data as possible about key measures of process/outcomes using integrated modes/channels of communication in a way that promotes use of the targeted innovation.* | RT, SD, SP | A clinical process measure is collected from clients and relayed via SP and is used clinically in each session. The measure is a weekly score of parents’ own targets with the intervention, which they score based on how close they perceive themselves to be to target goal. | (*Individual target*)  SP, clients (parents).  (*Conceptual target*) In addition to being a process/outcome measure in the study, the measure is an important tool in session with clients. It is used as a “compass” to check in whether clients are on track, and as such is used for planning and tailoring steps ahead. | Collected weekly, in beginning of each session. Data is then relayed electronically to RT each week. | Around 2-5 minutes with parents at the start of each session. Then about 5 minutes of relaying information electronically. | Fidelity, appropriateness. | (*Theoretical*)  (3) |  |  |
| **Engage consumers (n = 1)** | | | | | | | | | |  |  |
| 26 | *Intervene with patients/consumers to enhance uptake and adherence* | *Develop strategies with patients to encourage and problem solve around adherence.* |  | *Multifaceted strategy* | (*Conceptual target*) Acceptance, adherence, and engagement from end users of intervention is key. |  |  | Fidelity, appropriateness. | (*Theoretical*) (3)  (*Empirical*) SD, SS and SP extended experience with using active teaching strategies and home assignments in other EBIs. |  |  |
|  |  |  | RT | 26a) clients receive financial compensation (gift cards) to take part in study. | (*Individual target*)  Clients (parents) | Sent out each time data is collected from clients. Five timepoints, over a period of approx. one year. | Five gift cards, each equivalent of NOK200 (approx. USD19). |  |  |  |  |
|  |  |  | RT, SP | 26b) coordinators in RT are in regular contact with and remind clients to fill out measures. SP remind clients to attend sessions. | (*Individual target*)  Clients (parents) |  |  |  |  |  |  |
|  |  |  | SP | 26c) feedback regarding clients’ understanding and use of SPARCK is integrated into sessions and clients are explicitly invited to take part in formulating treatment goals. | (*Individual target*)  Clients (parents) |  |  |  |  |  |  |
|  |  |  | SP | 26d) an integral part of SPARCK is the use of active and dynamic teaching strategies in sessions, particularly the use of role play. | (*Individual target*)  Clients (parents) | In each session. |  |  |  |  |  |
|  |  |  | SP | 26e) an important element/process tool of the intervention is the use of home assignments between sessions, where parents try out strategies with the target child. | (*Individual target*)  Clients (parents) | Between each session. | One home assignment between sessions. |  |  |  |  |

**^a^SPARCK optimization study** refers to a previous mixed-methods study in seven municipalities, where SP in the Lab participated. SPARCK went through two cyclical tests of change, followed by stakeholder interviews and meetings for feedback. Prior to training of SP and initiation of current study, the changes to intervention and implementation strategies (e.g., session sequencing and supervision dosage) were applied. Data was also used when adapting IDM for the current study.

**^b^Municipality workshops**: Five regional workshops were held digitally, directed at all recruited municipalities, prior to training of SP. Municipalities were grouped together regionally, and breakout rooms were created for each municipality. Themes concerned information about the intervention, design of study, target groups, problem area, municipalities’ existing interventions for target group etc. Each workshop lasted 2.5 hours, was recorded and made available in a Microsoft Teams group.

**^c^National implementation team**: NIT is an established implementation team of two other EBIs (PMTO and TIBIR), consisting of five regional implementation teams representing all Norwegian health regions, as well as a central team. The regional members have established links to the municipalities where the current study takes place. Of the 14 SPARCK supervisors (SS), 11 are regional members of NIT, and they supervise their region’s SPARCK practitioners (SP). The four SD and one in the RT are central members, in addition to four who do not take part in the SPARCK study. NIT meets five to six times a year, in two-day meetings (10-12 days). After the current study, NIT will facilitate and support SPARCK implementation in municipalities.

**^d^The Lab seminars**: Seminars with the Lab, to give add-on training, tap into experiences with using SPARCK, descriptions of usual care, local needs. Mix of training, information sharing, group tasks and focus group interview.

**^e^The Lab**: The first wave SP (14) from seven municipalities who took part in SPARCK study 1. They took part in co-creation of the intervention and implementation strategies and their experiences from practicing and implementing SPARCK are documented. Practitioners in the Lab are experienced in using SPARCK, have had minimum two clinical cases each, and receives separate add-on training to the other SP.

**^f^Delphi study**: After the current study, a follow-up study will commence, as a modified Delphi survey among relevant stakeholders and implementation experts, to solicit and distill experts’ judgements on suitable implementation strategies to address barriers found in current study. Relevant theory and data from the SPARCK studies will guide the choice of strategies the participants are presented with. Survey will have two or three-rounds, each round taking participants 30-45 min. Based on this, implementation strategies for real-world implementation will be chosen, adapted, and deployed.

**^g^SPARCK implementation plan**: A formal implementation blueprint of SPARCK is initiated and will be a dynamic document that will be populated with data and experiences from the current study as it progresses. Each municipality will have their own version of the implementation plan. In the municipality workshops, all municipalities are asked to start making a draft for a municipal implementation plan for the SPARCK study and send to us after the workshop. They are given a template and asked to discuss and fill out a plan for recruitment of clients to RCT, overview of existing interventions for target group (possible treatments in control arm of RCT), name of contact person and local coordinator for current study, as well as plan for dissemination of information about the RCT and implementation study. If SPARCK clinical effect sizes are satisfactory and it is decided that SPARCK will be implemented in regular practice, implementation strategies will be further investigated, and the formal blueprint will be finalized in collaboration with stakeholders. The plan will be updated as needed and used actively to guide the implementation at each local site.

**^h^Electronic quality monitoring system**: NUBU has a pre-existing system for other EBIs which municipalities are familiar with, and all SP and SS use. Currently, SPARCK manuals, training materials, parent materials and formal information are available here. For other EBIs, the system enables tracking and monitoring of training and supervision, tracking of clinical and implementation activities and outcomes, and generation of graphical representation and reports. The system is a source for audit and feedback and for tracking implementation fidelity. The functionality for collection and monitoring of clinical and implementation activities and outcomes can be adapted and included for SPARCK in the future, as SPARCK clinical and implementation activities are comparable to the existing EBIs in the system.

**^i^SPARCK manuals and materials**: Detailed manuals for SP were developed and refined over years, with incorporation of changes made after SPARCK optimization study. Materials include handbook with background, theory of change, target groups, core components, sequencing, step-by-step descriptions of activities in sessions with clients, as well as short versions of handbook themes, tools and supporting materials, separate materials for clients (parents and child), as well as detailed training educational materials (e.g., PowerPoints, handouts, video recordings), which will enable others to perform training. Process skills and delivery methods of content is thoroughly addressed. Careful balancing between complex theoretical components and making the material pedagogical and easy-to-use was of high priority. Feedback and evaluation from the Lab in this process was crucial.

**^j^Implementation team infrastructure**: This infrastructure was established around 20 years ago, with national (NIT), regional, and local (municipal) teams implementing EBIs (PMTO and TIBIR). The national team supports regional teams, who are responsible for developing and supporting local implementation teams in each region. The local teams do the practical implementation in their own municipality. Each region has annual seminars for coordinators of local implementation teams, where experiences are shared, and common challenges are worked with. SPARCK can be included in this infrastructure.

**^k^NUBU core group**: The group responsible for the development of the SPARCK intervention, and work closely together in the current trial. The group consists of six researchers (RT) and four clinical experts (SD) with defined and clear roles. RT and SD developed SPARCK together, where RT are responsible for the research and study design and SD are responsible for the clinical intervention. The group has weekly/biweekly meetings, where RT and SD collaborate and problem solve on issues regarding the study (related to research, clinical, implementation, administration etc.).

### List of abbreviations

CFIR – Consolidated Framework for Implementation Research

EBI – Evidence based intervention

ERIC – Expert Recommendations for Implementing Change compilation

IDM – Implementation Determinants Measure

ML – Municipal leaders (study participants)

NIT – National implementation team

NUBU – Norwegian Center for Child Behavioral Development (Norwegian acronym)

ORIC – Organizational Readiness for Implementing Change

PARIHS – Promoting Action on Research Implementation in Health Services framework

RCT – Randomized controlled trial

RT – Research team, part of NUBU core group

SD – SPARCK developers; expert clinicians. Part of NUBU core group

SPARCK – Supportive Parents – Coping Kids

SP – SPARCK practitioners (study participants)

SS – SPARCK supervisors

T1 – early, after training of SP and information dissemination to ML

T2 – mid, after one year

T3 – late, after two years

**References**

1. Tømmerås T, Backer-Grøndahl A, Høstmælingen AT, Laland H, Gomez MB, Apeland A, et al. Study protocol for a randomized controlled trial of supportive parents – coping kids (SPARCK) - a transdiagnostic and personalized parent training intervention to prevent childhood mental health problems, BMC Psychology. 2024;12(1):264.

2. Proctor EK, Powell BJ, McMillen JC. Implementation strategies: recommendations for specifying and reporting. Implementation Science. 2013;8(1):139.

3. Powell BJ, Waltz TJ, Chinman MJ, Damschroder LJ, Smith JL, Matthieu MM, et al. A refined compilation of implementation strategies: results from the Expert Recommendations for Implementing Change (ERIC) project. Implementation Science. 2015;10(1):1-14.

4. Waltz TJ, Powell BJ, Matthieu MM, Damschroder LJ, Chinman MJ, Smith JL, et al. Use of concept mapping to characterize relationships among implementation strategies and assess their feasibility and importance: results from the Expert Recommendations for Implementing Change (ERIC) study. Implementation Science. 2015;10(1):109.

5. Proctor E, Silmere H, Raghavan R, Hovmand P, Aarons G, Bunger A, et al. Outcomes for implementation research: conceptual distinctions, measurement challenges, and research agenda. Administration and policy in mental health and mental health services research. 2011;38(2):65-76.

6. Tømmerås T, Backer-Grøndahl A, Arnesen A, Apeland A, Laland H, Askeland E, et al. Study Protocol for a Research and Development Project: Optimizing a Unified Parent Training Intervention to Prevent Child Mental Health Problems and Neglect. medRxiv. 2022.

7. Grønlie AA, Backer-Grøndahl A, Nes RB, Tømmerås T. Barriers and facilitators to implementing the preventive parent training intervention Supportive Parents - Coping Kids (SPARCK): a contextual analysis [Manuscript in preparation]. 2024.

8. Askeland E, Forgatch MS, Apeland A, Reer M, Grønlie AA. Scaling up an Empirically Supported Intervention with Long-Term Outcomes: the Nationwide Implementation of GenerationPMTO in Norway. Prevention Science. 2019;20(8):1189-99.

9. Damschroder LJ, Aron DC, Keith RE, Kirsh SR, Alexander JA, Lowery JC. Fostering implementation of health services research findings into practice: a consolidated framework for advancing implementation science. Implementation Science. 2009;4(1):50.

10. Damschroder LJ, Reardon CM, Widerquist MAO, Lowery J. The updated Consolidated Framework for Implementation Research based on user feedback. Implementation Science. 2022;17(1):75.

11. Shea CM, Jacobs SR, Esserman DA, Bruce K, Weiner BJ. Organizational readiness for implementing change: a psychometric assessment of a new measure. Implementation science. 2014;9(1):1-15.

12. Weiner BJ. A theory of organizational readiness for change. Implementation Science. 2009;4(1):67.

13. Beidas RS, Dorsey S, Lewis CC, Lyon AR, Powell BJ, Purtle J, et al. Promises and pitfalls in implementation science from the perspective of US-based researchers: learning from a pre-mortem. Implementation Science. 2022;17(1):55.

14. Waltz TJ, Powell BJ, Fernández ME, Abadie B, Damschroder LJ. Choosing implementation strategies to address contextual barriers: diversity in recommendations and future directions. Implementation Science. 2019;14(1):42.

15. Kirchner JE, Smith JL, Powell BJ, Waltz TJ, Proctor EK. Getting a clinical innovation into practice: An introduction to implementation strategies. Psychiatry Research. 2020;283:112467.

16. Harvey G, Kitson A. PARIHS revisited: from heuristic to integrated framework for the successful implementation of knowledge into practice. Implementation Science. 2016;11(1):33.

17. Nadeem E, Gleacher A, Beidas RS. Consultation as an Implementation Strategy for Evidence-Based Practices Across Multiple Contexts: Unpacking the Black Box. Administration and Policy in Mental Health and Mental Health Services Research. 2013;40(6):439-50.

18. May CR, Johnson M, Finch T. Implementation, context and complexity. Implementation Science. 2016;11(1):1-12.

19. Fixsen DL, Naoom SF, Blase KA, Friedman RM, Wallace F, Burns B, et al. Implementation research: A synthesis of the literature. 2005.

20. von Thiele Schwarz U, Aarons GA, Hasson H. The Value Equation: Three complementary propositions for reconciling fidelity and adaptation in evidence-based practice implementation. BMC Health Services Research. 2019;19(1):868.

21. Miech EJ, Rattray NA, Flanagan ME, Damschroder L, Schmid AA, Damush TM. Inside help: An integrative review of champions in healthcare-related implementation. SAGE Open Medicine. 2018;6:2050312118773261.
