# Supplementary material for: Implementation of a parent training intervention (SPARCK) to prevent childhood mental health problems: study protocol for a pragmatic implementation trial in Norwegian municipalities
Source: Trials. 2024 Dec 21;25:846. doi: 10.1186/s13063-024-08704-7 (PMC11663316; doi:10.1186/s13063-024-08704-7)
Supplement: Supplementary file 2 — Additional file 2: TIDieR checklist. [file 13063_2024_8704_MOESM2_ESM.docx]

**
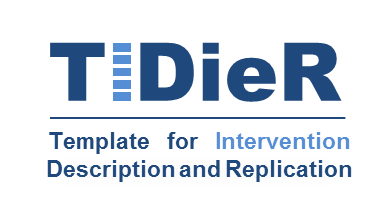
The TIDieR (Template for Intervention Description and Replication) Checklist*:**

Information to include when describing an intervention and the location of the information

| **Item number** | **Item TIDieR checklist for the SPARCK intervention** | **Where located **** | |
| --- | --- | --- | --- |
|  |  | Primary paper  (page or appendix  number) | Other ^†^ (details) |
|  | **BRIEF NAME** |  |  |
| **1.** | Supportive Parents – Coping Kids (SPARCK). | ______5_______ | ____________ |
|  | **WHY** |  |  |
| **2.** | SPARCK is a transdiagnostic parent training intervention aimed at children (4 to 12 years) with elevated but sub-clinical levels of internalizing and/or externalizing symptoms, and contains well-established elements/strategies, which are tailored to help parents promote coping skills in their children (page 5). The goal is to reduce mental health problems in children and promote positive parent-child interactions. Most children and families in Norway do not have access to EBIs, therefore SPARCK is directed at the frontline municipal level and co-created with relevant stakeholders to increase probability of adoption, implementation, and sustainment (pages 3-4). | ______3-5______ | (also see protocol for RCT study [1]) |
|  | **WHAT** |  |  |
| **3.** | Materials: A detailed manual for SPARCK practitioners to be used in intervention delivery has been developed and distributed to SPARCK practitioners. Manual includes background, ToC, target groups, core components, sequencing, short versions, supporting materials, as well as separate materials for clients (parents and child). All practitioners have access to materials in an electronic decision support data system which requires a login. All materials related to the training of SPARCK practitioners are also available in the data system. Other information regarding the study to participants and leaders, including reminders, nudges, clarifications, answers to questions etc., are available either via e-mails and/or a Microsoft Teams group. | Additional file 1 | (1) |
| **4.** | Procedures: Prior to initiation of study, the intervention was developed, piloted, underwent cyclical tests of change followed by optimization together with municipal stakeholders (pages 5-7). The first test cycle occurred during the corona shutdown in Norway in 2020, leading to changes in some procedures and supporting materials, such as delivery mode from face-to-face to video call or telephone (see reference 2). Delivery mode was reversed back to face-to-face after shutdown period was over, following feedback from clients and participants. Prior to current study, all instruments in the current study have been translated, adapted to context, and piloted (page 12). All other initiated or planned implementation strategies related to the current study, as well as the RCT, are described in Additional file 1. Other procedures related to the delivery of the intervention to clients, are described in RCT protocol (see reference 1). | __5-7, and Additional file 1 | (1, 2 (on clients’, who received SPARCK, perceptions on effects of COVID-19 pandemic), protocol for intervention development [3], 4) |
|  | **WHO PROVIDED** |  |  |
| **5.** | There are two participant categories, SPARCK practitioners, who provide the intervention to the clients, and municipal leaders. A total of 24 municipalities have been recruited to the study, and a total of 44 practitioners will be trained in the intervention. Practitioners are employed in municipal frontline mental health services, such as health care, school health services, and child welfare services, where they work as clinicians with the target group. Of the 44, 14 of the practitioners (labelled “the lab”) were trained in SPARCK in two iterative mixed-methods cycles (2020-2021), in which they tested the intervention with client families. They contributed to co-creation of SPARCK and are more experienced in using the intervention. For the current study, they receive add-on training which reflects the changes made to the intervention since they performed it. In addition to SPARCK, all practitioners are trained in and actively practice Parent Management Training – Oregon model (PMTO) (see references 5 and 6). Participants also include the practitioners’ leaders, including their immediate manager, and superior leaders at mid and top managerial levels in the various services. Demographic and service-related variables will be obtained from all participants (i.e., type of employment service, educational level, professional background, role, job experience, training in EBIs, counselling experience, familiarity with SPARCK etc.). | __10, 11-- | _ (1-3, 5, 6) |
|  | **HOW** |  |  |
| **6.** | Descriptions of delivery modes of the intervention can be found in the RCT protocol (reference 1). SPARCK will be delivered as an individual intervention, face-to-face. It has previously been delivered digitally by “the lab”, due to corona restrictions (reference 2), and such exceptions can be made if necessary. Sessions should ideally be attended by both parents. Parents are given the option of bringing the child to sessions. | _____5______ | ___ (1, 2)___ |
|  | **WHERE** |  |  |
| **7.** | Clients will be recruited from 24 Norwegian municipalities, which vary in size, demography and urbanicity. All five official health regions are represented. Efforts were made to recruit heterogeneous municipalities, to reflect conditions as they are in the real world. SPARCK practitioners will recruit cases to the study via the municipalities’ frontline mental health services’ referral system, which will vary somewhat across sites. The services where the practitioners are employed include health care, school health services, and child welfare. The intervention sessions will be delivered at the location of the various service agency facilities, in rooms suited and equipped for working with clients. | _____11______ | ____ (1)____ |
|  | **WHEN and HOW MUCH** |  |  |
| **8.** | The intervention will be delivered weekly in up to 12 sessions. Some cases will need less than 12, however, practitioners are instructed to deliver a maximum of 12 sessions. Each session will last approximately one hour at timepoints that suit the families. In addition to the 12 consecutive sessions, each family are offered the opportunity to get one booster session three months after intervention is terminated. See further descriptions in RCT protocol (reference 1). During the study, we will collect information and report on number of sessions, duration, and dose. | Page 5, Additional file 1 | ____ (1)____ |
|  | **TAILORING** |  |  |
| **9.** | See RCT protocol for thorough descriptions of intervention and figure of Theory of Change (reference 1). Clients allocated to the intervention arm, will receive an intervention tailored to their needs and symptomatology. Clients in Treatment as Usual arm will not be addressed in the current study. In the first SPARCK session, practitioners and parents do a “mapping”, which is central for the customization of intervention components according to the families’ needs. During this session, parents collaborate with practitioners to make clear and concise goals for the intervention. After mapping, there are four distinct, “recommended courses” of SPARCK intervention, which each give guidelines for choice of specific SPARCK components. Choice of components are based on levels of child symptoms on anxiety, depression, and conduct problems, as well as parental caregiving challenges, in addition to the parents’ own goals for the intervention. Choices and decision points are made by the practitioners, closely guided by clinical supervision and expert consultation. The four recommended courses provide the SPARCK practitioners with both structure and flexibility to balance the need for a clear direction with the need to personalize the content. This was one of the most important take-home-message from “the lab”, when they took part in the co-creative optimization of SPARCK (reference 3). | Page 5, Additional file 1 | ___ (1, 3)___ |
|  | **MODIFICATIONS** |  |  |
| **10.^ǂ^** | If the intervention was modified during the course of the study, describe the changes (what, why, when, and how). | N/A (protocol) | __________ |
|  | **HOW WELL** |  |  |
| **11.** | Planned: New SPARCK practitioners were gathered as one group and received training in the intervention prior to study initiation. Training lasted for five days with a focus on interactive and dynamic training techniques, such as use of demonstrations and role play, both live and videotaped, in addition to psychoeducation. All sessions were videotaped and made available to practitioners, should they need to repeat or if additional SPARCK practitioners were to be included in trial at a later point. The SPARCK practitioners who had participated in the optimization trial of SPARCK (reference 3) received separate add-on training prior to current study, delivered jointly via an online video call. This training was also videotaped. All training materials are tangible documents which are available to all SPARCK practitioners and supervisors. All SPARCK practitioners attend clinical supervision throughout the duration of the trial, provided weekly during their first two cases, which switches to biweekly from case number three and onwards. Supervision is group-based and provided via online video calls with 1-3 other practitioners. In each session, each SPARCK practitioners gets around 30 minutes to discuss their case with an experienced SPARCK supervisor. In addition, each supervisor attends weekly or biweekly (according to needs) consultation (“supervision-of-supervisor”), with a member of the SPARCK developer team. Supervision-of-supervisor is also group-based and provided via online video calls with 2-4 other practitioners. maintain fidelity, substantial efforts have gone into the production and refinement of materials and tools for the practitioners. The practitioners’ adherence to the intervention will be measured throughout the trial via a fidelity questionnaire (FidQ). The FidQ details recipients, intervention strategies employed, pedagogical tools, client engagement, and parental assessments of goals in intervention. The measure will be completed by all SPARCK practitioners weekly after each session. | Pages 11-12, Additional file 1 | ___ (1, 3)___ |
| **12.^ǂ^** | Actual: If intervention adherence or fidelity was assessed, describe the extent to which the intervention was delivered as planned. | N/A (protocol) | __________ |

** **Authors** - use N/A if an item is not applicable for the intervention being described. **Reviewers** – use ‘?’ if information about the element is not reported/not sufficiently reported.

† If the information is not provided in the primary paper, give details of where this information is available. This may include locations such as a published protocol or other published papers (provide citation details) or a website (provide the URL).

ǂ If completing the TIDieR checklist for a protocol, these items are not relevant to the protocol and cannot be described until the study is complete.

* We strongly recommend using this checklist in conjunction with the TIDieR guide (see *BMJ* 2014;348:g1687) which contains an explanation and elaboration for each item.

* The focus of TIDieR is on reporting details of the intervention elements (and where relevant, comparison elements) of a study. Other elements and methodological features of studies are covered by other reporting statements and checklists and have not been duplicated as part of the TIDieR checklist. When a **randomised trial** is being reported, the TIDieR checklist should be used in conjunction with the CONSORT statement (see [www.consort-statement.org](http://www.consort-statement.org)) as an extension of **Item 5 of the CONSORT 2010 Statement.** When a **clinical trial** **protocol** is being reported, the TIDieR checklist should be used in conjunction with the SPIRIT statement as an extension of **Item 11 of the SPIRIT 2013 Statement** (see [www.spirit-statement.org](http://www.spirit-statement.org)). For alternate study designs, TIDieR can be used in conjunction with the appropriate checklist for that study design (see [www.equator-network.org](http://www.equator-network.org)).

**References**

1. Tømmerås T, Backer-Grøndahl A, Høstmælingen AT, Laland H, Gomez MB, Apeland A, et al. Study protocol for a randomized controlled trial of supportive parents – coping kids (SPARCK) - a transdiagnostic and personalized parent training intervention to prevent childhood mental health problems, BMC Psychology. 2024;12(1):264.
2. Backer-Grøndahl A, Arnesen A, Idsøe T, Grønlie AA, Tømmerås T. Forebyggende hjelpetiltak under korona våren 2020. Psykologi i kommunen. 2022;1.
3. Tømmerås T, Backer-Grøndahl A, Arnesen A, Apeland A, Laland H, Askeland E, et al. Study Protocol for a Research and Development Project: Optimizing a Unified Parent Training Intervention to Prevent Child Mental Health Problems and Neglect. medRxiv. 2022.
4. Grønlie AA, Backer-Grøndahl A, Nes RB, Tømmerås T. Barriers and facilitators to implementing the preventive parent training intervention Supportive Parents - Coping Kids (SPARCK): a contextual analysis [Manuscript in preparation]. 2024.
5. Askeland E, Forgatch MS, Apeland A, Reer M, Grønlie AA. Scaling up an Empirically Supported Intervention with Long-Term Outcomes: the Nationwide Implementation of GenerationPMTO in Norway. Prevention Science. 2019;20(8):1189-99.
6. Forgatch MS, Patterson GR. Parent Management Training—Oregon Model: An intervention for antisocial behavior in children and adolescents. In: Weisz JR, Kazdin AE, editors. Evidence-based psychotherapies for children and adolescents: Guilford Press; 2010.
